# Efficacy of 5-aminolevulinic acid-mediated photodynamic therapy using light-emitting diodes in human colon cancer cells

**DOI:** 10.3892/or.2013.2220

**Published:** 2013-01-03

**Authors:** TOMOYA HATAKEYAMA, YASUTOSHI MURAYAMA, SHUHEI KOMATSU, ATSUSHI SHIOZAKI, YOSHIAKI KURIU, HISASHI IKOMA, MASAYOSHI NAKANISHI, DAISUKE ICHIKAWA, HITOSHI FUJIWARA, KAZUMA OKAMOTO, TOSHIYA OCHIAI, YUKIHITO KOKUBA, KATSUSHI INOUE, MOTOWO NAKAJIMA, EIGO OTSUJI

**Affiliations:** 1 Division of Digestive Surgery, Department of Surgery, Kyoto Prefectural University of Medicine, Kamigyo-ku, Kyoto 602-8566, Japan; 2 SBI Pharmaceuticals Co., Ltd., Minato-ku, Tokyo 106-6019, Japan

**Keywords:** 5-aminolevulinic acid, photodynamic therapy, colon cancer, light-emitting diode, protoporphyrin IX

## Abstract

5-Aminolevulinic acid (ALA)-mediated photodynamic therapy (PDT) (ALA-PDT) is a highly selective treatment for malignant cells. ALA-PDT has the potential to develop into a novel therapeutic strategy for various types of cancer. Recently, light-emitting diodes (LEDs), which are inexpensive, stable and easier to handle compared to lasers, have been used in PDT as a light source. However, in colorectal cancer (CRC), the efficacy of ALA-PDT in combination with LEDs has not been fully assessed. Therefore, in this study, we evaluated the antitumor effect of ALA-PDT using various LEDs in colon cancer cells. The HT-29 human colon cancer cell line was used both *in vitro* and *in vivo*. HT-29 cells were seeded in 96-well plates. Following 5-ALA administration, cells were irradiated using LEDs at different wavelengths. Three types of LEDs, blue (peak wavelength, 456 nm), white (broad-band) and red (635 nm) were used. Twenty-four hours after irradiation, the cytotoxic effects of ALA-PDT were measured using the 3-(4,5-dimethylthiazol-2-yl)-2,5-diphenyltetrazolium bromide (MTT) assay. In order to evaluate the antitumor effect of ALA-PDT *in vivo*, nude mice were inoculated with HT-29 cells. Xenograft mice were injected intraperitoneally with 5-ALA and irradiated with 3 types of LEDs at a measured fluence rate of 96 mW/cm^2^ and fluence of 32 J/cm^2^. Each group comprised 6 mice. ALA-PDT was repeated 3 times at weekly intervals. Tumor weights were measured. Compared to the controls, ALA-PDT using LEDs showed significant antitumor effects *in vitro* and *in vivo*. The blue and white LEDs demonstrated greater antitumor effects compared to the red LEDs *in vitro* and *in vivo*. In particular, tumor inhibition rates in the blue and white LED groups were approximately 88% to those of the control group in the mouse models. In conclusion, ALA-PDT using LEDs is effective and useful in the treatment of CRC cells. This method could be a novel treatment modality for CRC.

## Introduction

Colorectal cancer (CRC) is the third most common cancer and the fourth most common cause of cancer-related mortality worldwide. The most common curative therapy for colon cancer is surgical resection. The survival rate of CRC patients correlates with tumor stage and the 5-year relative survival is approximately 65% (1). However, patients with advanced disease with unresectable metastatic lesions have a 5-year survival of approximately 5% (1,2). Several issues remain unsolved. For instance, effective treatment modalities are rare in cases of patients with peritoneal carcinomatosis, chemotherapy-resistant tumors and poor performance status. Therefore, novel treatment modalities for such cases are required.

Photodynamic therapy (PDT) was introduced approximately 35 years ago. PDT consists of: systemic or topical administration of a photosensitizer or metabolic precursor, photoexcitation of the sensitizer by light in the visible wavelength (400–750 nm) and tumor cell death induced by the release of reactive oxygen species (ROS) (3,4). PDT provides better selectivity for the targeting of tumors compared to conventional chemo-and radiotherapy due to the preferential accumulation of photosensitizers in tumors (3,4). Photofrin^®^ (Porfimer sodium) has received worldwide regulatory approval as a photosensitizer and was the basis for the growth of oncologic PDT (3,4). However, Photofrin requires approximately 6 weeks of photosensitivity precautions (4). This side-effect has limited its use as a photosensitizer for PDT.

Protoporphyrin IX (PpIX), synthesized from 5-aminolevulinic acid (ALA) in the mitochondria, is an intrinsic and safe photosensitizer (3–5). PpIX accumulates in several malignant tumors following 5-ALA administration. This phenomenon is widely applicable for photodynamic diagnosis (PDD) and PDT (3–5). Recently, we reported the utility of lymph node metastasis detection using 5-ALA administration in mouse models (6). One of the main advantages of ALA-PDT is that PpIX is cleared from the body within 24–48 h subsequent to systemic ALA administration (4). Furthermore, 5-ALA is an endogenous agent that is part of the regular diet. Therefore, ALA-PDT has the potential to avoid the risk of prolonged phototoxicity associated with the conventional photosensitizer, Photofrin (4).

ALA-PDT has been performed using lasers as light sources at 635 nm. However, since lasers are large, complex and expensive, PDT has not been widely used in clinical treatment. Over the past decade, light-emitting diodes (LEDs), which are inexpensive, stable, easy to operate, require little maintenance and provide wide area illumination fields, have been used in PDT instead of lasers (7,8). Therefore, ALA-PDT using LEDs has the potential to rapidly become a useful treatment modality.

ALA-PDT is non-invasive and may be used in repeated treatments and in combination with surgery, chemo- and radiotherapy or other modalities. Over the past decade, investigators have reported the effects of ALA-PDT in various tumor cells (9–16). In dermatology, multicenter randomized controlled studies have demonstrated the high efficacy of topical ALA-PDT for actinic keratosis, Bowen’s disease and superficial basal cell carcinoma (17–19). Although certain reports have addressed the efficacy of ALA-PDT in CRC cells (20,21), these were limited to *in vitro* studies. Therefore, the objective of this study was to investigate the antitumor effect of ALA-PDT using LEDs for the treatment of CRC cells in a xenograft mouse model.

## Materials and methods

### Cell line and cell culture

The HT-29 human CRC cell line was purchased from the American Type Culture Collection (Rockville, MD, USA). HT-29 cells were grown in McCoy’s medium with 10% fetal bovine serum (FBS), 100 U/ml penicillin and 100 μg/ml streptomycin at 37°C in a water-saturated atmosphere with 5% CO_2_/95% air.

### Animals

Four-week-old female BALB/c mice were used in this study. The mice were housed in groups, in plastic cages with stainless-steel grid tops in an air-conditioned environment with a 12-h light-dark cycle and were provided with food and water *ad libitum*. The animal experiments were conducted in accordance with the institutional guidelines of the Kyoto Prefectural University of Medicine, Kyoto, Japan.

### Cancer-bearing mouse model

**Figure 1:**
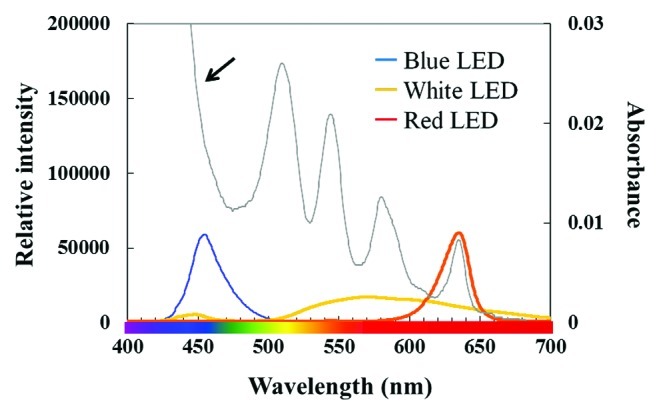
Correlation between the absorption spectrum of protoporphyrin IX (PpIX) solution and exposure spectrum of the 3 types of light-emitting diodes (LEDs). PpIX has several peaks in the absorption spectrum (arrow). The blue band is markedly larger compared to the absorption band at 635 nm.

HT-29 cells (0.5×10^6^) were inoculated subcutaneously in 100 μl of phosphate-buffered saline (PBS) into the flanks of nude mice under general anesthesia. One week later, the longest diameter of the xenograft tumor was between 3 and 5 mm. The mice were then divided into a treatment and a control group. The treatment group consisted of 3 subgroups: the blue LED (peak wavelength, 456 nm), the white LED and the red LED (635 nm) groups (Fig. 1). These LED irradiation units were provided by SBI Pharmaceuticals Co., Ltd. (Tokyo, Japan). The control group and treatment subgroups comprised 6 mice each.

### Light sources for photodynamic therapy

Cultured cell plates and inoculated mice were exposed to the 3 types of LED lights (Fig. 1). Light intensity was measured using a photo-radiometer.

### Photodynamic therapy in vitro

HT-29 cells (5×10^4^ cells/0.1 ml) were seeded in 96-well plates and placed in an incubator at 37°C for 24 h. The medium was then replaced with medium containing 1 mM 5-ALA (Cosmo Bio International, Tokyo, Japan) (6,15). Three hours later, the 5-ALA-containing medium was replaced with PBS. Cells were irradiated with 3 types of LED at a measured fluence rate of 16 mW/cm^2^ and fluence of 3.0 J/cm^2^. PBS was replaced with fresh medium immediately subsequent to irradiation. The control group was not exposed to ALA administration or LED irradiation. Twenty-four hours later, cell viability was determined using the 3-(4,5-dimethylthiazol-2-yl)-2,5-diphenyltetrazolium bromide (MTT) assay (15). Sample absorbances were read on a MaxLine Microplate Reader equipped with a 550-nm filter. Five separate experiments were performed.

### Photodynamic therapy in vivo

Nude mice in the treatment group received an intraperitoneal injection of 250 mg/kg of 5-ALA (6,16). Five hours later, mice were irradiated with LEDs at a measured fluence rate of 96 mW/cm^2^ and fluence of 32 J/cm^2^. The 3 types of LEDs described above were used in this study. The control group was not exposed to ALA administration or LED irradiation. ALA-PDT was repeated 3 times at weekly intervals. Three weeks after the initial treatment, the mice were sacrificed under general anesthesia and tumors were removed (16). Removed tumor weights were measured (16).

### Statistical analysis

Differences in tumor weight and cell viability among the groups were analyzed using the non-parametric Mann-Whitney U test. P<0.05 was considered to indicate a statistically significant difference.

## Results

### Efficacy of ALA-PDT using LEDs for the treatment of CRC in vitro

**Figure 2:**
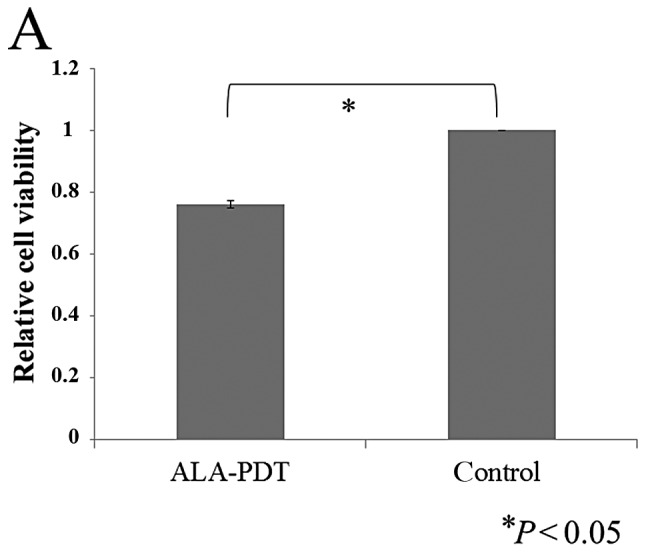

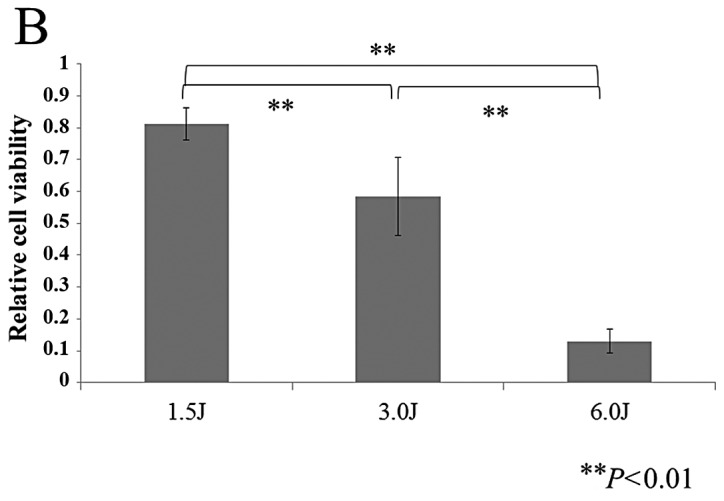
(A) Cell viability of HT-29 cells with 5-aminolevulinic acid (ALA)-mediated photodynamic therapy (PDT) (ALA-PDT), using red LEDs (peak wavelength, 635 nm; fluence, 3 J/cm^2^) was significantly lower compared to the control group. (B) Increasing light doses resulted in significant reductions in cell viability (peak wavelength, 635 nm; fluence rate, 15.9 mW/cm^2^).

First, the efficacy of ALA-PDT in the HT-29 cell line was evaluated. Red LEDs (peak wavelength, 635 nm) were used in this study since 635 nm is applied for conventional ALA-PDT (4). The cell viability of HT-29 cells was significantly lower in the group treated with ALA-PDT using red LED compared to the control group (P<0.05) (Fig. 2A). Moreover, increasing light doses resulted in significant reductions in cell viability (Fig. 2B). These results indicate that ALA-PDT using red LEDs is effective in treating human colon cancer cells and that the antitumor effects are dependent on fluence.

### Types of LEDs that are most suitable for ALA-PDT in HT-29

**Figure 3:**
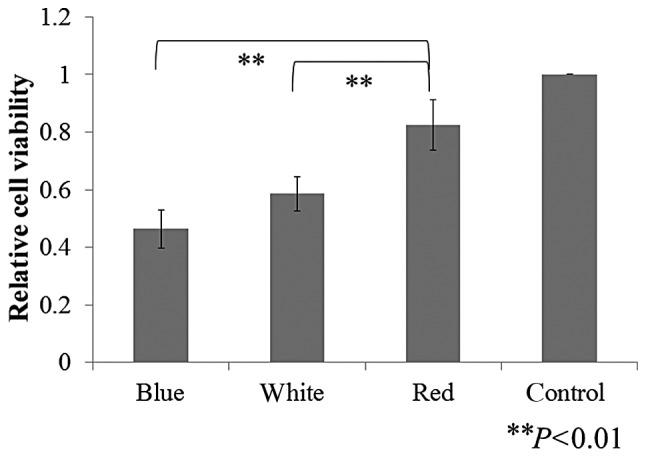
Comparison of the efficacy of ALA-PDT, using 3 types of LED in HT-29 cells. ALA-PDT with blue LEDs showed the greatest antitumor effect in the HT-29 cells.

Fig. 1 shows the correlation between the absorption spectrum for PpIX and emission spectrum of the 3 types of LEDs. For the absorption spectrum of PpIX, there is a maximum peak at 410 nm and smaller peaks near 510, 545, 580 and 630 nm. Therefore, we used blue (peak wavelength, 456 nm), white and red (635 nm) LEDs. Cell viability was significantly lower in the treatment groups compared to the control group (Fig. 3). The blue and white LEDs showed greater antitumor effects compared to the red LEDs (Fig. 3). This result indicates that blue and white LEDs are potential light sources for ALA-PDT in human colon cancer cells.

### Antitumor effect of ALA-PDT using LEDs in a CRC-bearing mouse model

**Figure 4:**
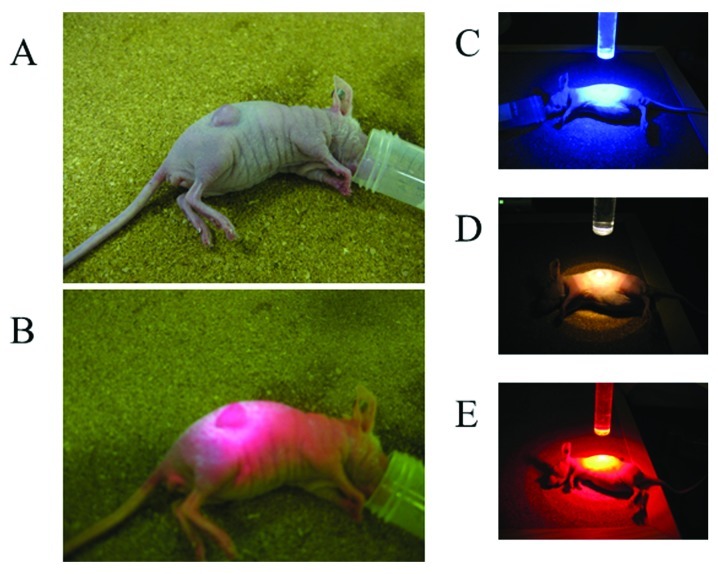
Photodynamic therapy *in vivo*. (A) HT-29-inoculated nude mouse. (B) Photodynamic detection of ALA-induced PpIX. (C) Photodynamic therapy using blue LEDs. (D) Photodynamic therapy using broad-band white LEDs. (E) Photodynamic therapy using red LEDs.

**Figure 5:**
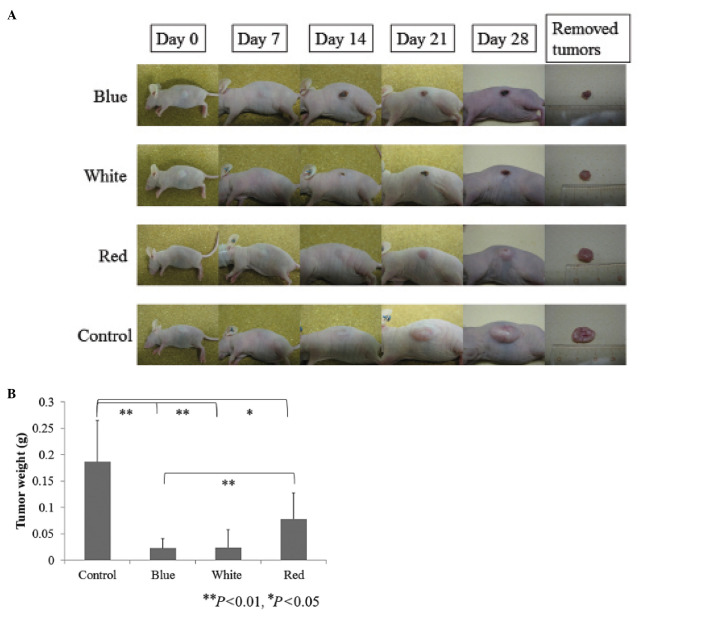
(A) Treatment progress of ALA-PDT using 3 types of LED in xenograft mouse models. ALA-PDT was repeated 3 times at weekly intervals. One week after the final irradiation, the mice were sacrificed and tumors were removed. Removed tumor weights were measured. (B) Comparison of tumor weights. The growth of HT-29 tumors was significantly slower in the treatment groups compared to the control group. Of the treatment groups, blue LEDs showed a greater antitumor effect compared to the conventional red LEDs.

Fig. 4 shows the experimental procedure of ALA-PDT using LEDs in a CRC-bearing mouse model. Five hours subsequent to 5-ALA administration, tumors were detected as red fluorescence using ALA-PDD (Fig. 4B). Tumor growth was clearly suppressed in the treatment groups (Fig. 5A). Tumor weights were significantly lower in the treatment groups compared to the control group (Fig. 5B). In the blue and white LED groups, the tumor inhibition rates were approximately 88% to those of the control group (Fig. 5B). Similar to the *in vitro* study, the antitumor effects of ALA-PDT using blue or white LEDs varied significantly from those with red LEDs (Fig. 5B). These results indicate that ALA-PDT using LEDs, particularly blue or white, is a potentially effective treatment modality for human colon cancer cells.

## Discussion

In this study, ALA-PDT using 3 types of LEDs demonstrated significant antitumor effects *in vitro* and *in vivo*. ALA-PDT using blue or white LEDs was more effective compared to the conventional red LEDs. These results suggest that ALA-PDT using LEDs, particularly blue or white, is a potential novel treatment modality for human CRC cells.

In general, lasers are used as light sources in ALA-PDT. Over the past decade, the efficacy of LEDs for ALA-PDT has been reported (7,9,22). In this study, we demonstrated the efficacy of LEDs as a light source for ALA-PDT in human colon cancer cells. Since LEDs have several advantages, such as being smaller, easier to use, and more cost-effective compared to lasers, they may soon be widely used as a novel light source for ALA-PDT in human colon cancer cells.

In the majority of cases where ALA-PDT is used for the treatment of various types of cancer, a laser emitting 630 nm is usually used (17–19,23). However, the most effective wavelength for ALA-PDT in human CRC cells has not yet been fully evaluated. Our *in vivo* study suggests that ALA-PDT using blue or white LEDs may be more suitable for the treatment of CRC cells compared to conventional red LEDs. The optimal wavelength for PDT should be chosen using an appropriate action spectrum. For PpIX, the Soret band (400–500 nm) is 20–30-fold larger compared to the absorption band at 630–635 nm (4). Therefore, blue light sources have been developed for this treatment. However, the absorption of hemoglobin and melanin, the main absorbers in human tissue, decreases with increasing wavelengths (24,25). In addition, the penetration depth of light into tissue increases with increasing wavelength, up to at least 800 nm (4). Light within the Soret band (410 nm) leads to the highest level of cell inactivation, up to approximately 2 mm from the surface in human skin and muscle tissues; at depths exceeding 2 mm, 635 nm light may be optimal (4,26). Therefore, we expected the blue LED to be less effective compared to the red LED in our *in vivo* study. However, we showed that ALA-PDT using blue LEDs yielded the greatest antitumor effects *in vitro* and *in vivo*. These results may mainly be due to the tumor specificity and higher absorption of PpIX in the blue band compared to that in the red band (Fig. 1). Another reason is that the blue LED may have photo-degraded PpIX as well as its photoproduct, photo-protoporphyrin (Ppp). Ppp has 2 absorption peaks at approximately 440 and 670 nm (27). Based on these absorption peaks, the combination of 2 wavelengths, 635 and 670 nm, or a broad spectral region covering the 2 peaks may be more effective compared to using a single wavelength (4). A previous report has demonstrated the efficacy of combining these 2 wavelengths in ALA-PDT (27). However, for Ppp, the blue band (at approximately 450 nm) is markedly larger compared to the absorption band at 670 nm (27). Therefore, irradiation by the blue LED (at approximately 450 nm) may effectively induce the photo-degradation of PpIX as well as that of Ppp, resulting in additional PDT effects. Consequently, ALA-PDT using blue LED had the most antitumor effect in our study. Notwithstanding, ALA-PDT using the white LED with a broad spectrum, demonstrated high efficacy, similar to the blue LED. This result may mainly be due to the broad spectrum that covers the peaks of PpIX and Ppp.

CRC is the third most common cancer and the fourth most common cause of cancer-related mortality worldwide. The most common curative therapy for colon cancer is surgical resection. The reported incidence of recurrent disease after primary curative resection ranges from 5 to 50% (28–30). Peritoneal carcinomatosis (PC) is common and is the second most frequent cause of mortality in CRC patients (31,32). For CRC patients with PC, the mean and median overall survivals have been reported to be 6.9 and 5.2 months, respectively (31). During the past decade, the development of a new concept involving cytoreductive surgery and hyperthermic intraperitoneal chemotherapy (HIPEC) has produced promising results (32). However, these procedures are so invasive that the rate of major morbidity and mortality is extremely high (32,33). Therefore, novel and non-invasive treatments for patients with PC are required. ALA-PDT using LEDs may have the potential to become a novel treatment for CRC patients with PC, since ALA-PDT is non-invasive and has the potential to be used in repeated treatments and in combination with other modalities.

The preferential accumulation of ALA-induced porphyrins in tumor cells provides the possibility of photo-detection by PpIX fluorescence (4,5,34). This procedure may be performed by means of fiber optic monitoring systems or fluorescence imaging systems after topical, local internal or systemic administration of ALA. A certain study demonstrated strong red fluorescence induced by ALA in urothelial carcinoma using fluorescence cystoscopy (34). Moreover, laparoscopy equipped with a fluorescence imaging system is used for PDD in urology (35,36). In gastrointestinal tumors, a certain study showed that laparoscopic fluorescence diagnosis using ALA administration may increase the sensitivity and specificity of diagnostic staging laparoscopy in rats with induced peritoneal dissemination (37). Over the past decade, laparoscopic-assisted colectomy (LAC) has been performed as standard surgery for CRC patients. Therefore, in the future, ALA-PDT and PDD using fluorescence laparoscopy may prove to be effective diagnostic and treatment modalities for CRC patients with PC.

Our study has inherent limitations since we used subcutaneous xenograft, not PC models and only one cell line, HT-29. Thus, additional studies are required to confirm whether or not ALA-PDT using LEDs is efficacious in other cell lines and in mice with PC.

In conclusion, we demonstrate that ALA-PDT using LEDs induces tumor cell death in the HT-29 CRC cell line *in vitro* and *in vivo*. Our findings provide insight into a novel treatment modality for CRC patients.
